# Clinical significance of cervical MRI in brachial plexus birth injury

**DOI:** 10.1080/17453674.2018.1562621

**Published:** 2019-01-23

**Authors:** Petra Grahn, Tiina Pöyhiä, Antti Sommarhem, Yrjänä Nietosvaara

**Affiliations:** a New Children’s Hospital, HUS Helsinki University Hospital, Department of Children’s Orthopedics and Traumatology, Helsinki;; b HUS Medical Imaging Center, HUS Helsinki University Hospital, Department of Radiology, Helsinki, Finland

## Abstract

Background and purpose — Patient selection for nerve surgery in brachial plexus birth injury (BPBI) is difficult. Decision to operate is mostly based on clinical findings. We assessed whether MRI improves patient selection.

Patients and methods — 157 BPBI patients were enrolled for a prospective study during 2007–2015. BPBI was classified at birth as global plexus injury (GP) or upper plexus injury (UP). The global plexus injury was subdivided into flail upper extremity (FUE) and complete plexus involvement (CP). Patients were seen at set intervals. MRI was scheduled for patients that had either GP at 1 month of age or UP with no antigravity biceps function by 3 months of age. Type (total or partial avulsion, thinned root), number and location of root injuries and pseudomeningoceles (PMC) were registered. Position of humeral head (normal, subluxated, dislocated) and glenoid shape (normal, posteriorly rounded, pseudoglenoid) were recorded. Outcome was assessed at median 4.5 years (1.6–8.6) of age.

Results — Cervical MRI was performed on 34/157 patients at median 3.9 months (0.3–14). Total root avulsions (n = 1–3) were detected on MRI in 12 patients (8 FUE, 4 CP). Reconstructive surgery was performed on 10/12 with total avulsions on MRI, and on all 10 with FUE at birth. Sensitivity and specificity of MRI in detecting total root avulsions was 0.88 and 1 respectively. Posterior shoulder subluxation/dislocation was seen in 15/34 patients (3.2–7.7 months of age).

Interpretation — Root avulsion(s) on MRI and flail upper extremity at birth are both good indicators for nerve surgery in brachial plexus birth injury. Shoulder pathology develops very early in permanent BPBI.

Neonatal brachial plexus injury (BPBI) occurs in 0.5–4/1,000 vaginal births (Hoeksma et al. 2000, Foad et al. 2008, Pöyhiä et al. 2010). Approximately 80% of these patients recover spontaneously during their first year of life (Pöyhiä et al. 2010). In patients with a permanent injury BPBI causes muscle changes in infants often leading to shoulder and elbow contractures (Eisman et al. 2015, Gharbaoui et al. 2015). Without treatment at least one-third of these develop posterior instability, subluxation, and deformity of the glenohumeral joint (Hoeksma et al. 2000, 2003, Moukoko et al. 2004). Patients with root avulsions have a poorer prognosis compared with patients without (Kirjavainen et al. 2007).

There is evidence that some of the patients with a permanent palsy benefit from surgical repair of the lesion (Waters 2005, Hale et al. 2010). The extent and the type of root injury can be evaluated by clinical, neurophysiological, and different radiographic methods. Different grading systems (Narakas classification, Gilbert shoulder and Gilbert–Raimondi classification, Active Movement Scale and 3-month Toronto Test Score, 9-month Cookie Test) (Narakas 1986, Curtis et al. 2002, Haerle and Gilbert 2004, Borschel and Clarke 2009, Bade et al. 2014) have been developed for prognostic purposes in an attempt to help in surgical decision-making. At present, however, there is no consensus regarding the indications and timing of surgery in BPBI.

We have prospectively studied the clinical significance of high-resolution cervical MRI in BPBI patients who were considered for brachial plexus surgery during a 9-year period from 2007 to 2015. Our hypothesis was that evidence of total root avulsion injury on MRI is a good indicator for surgical repair.

## Patients and method

During the study period between 2007 and 2015 altogether 157 BPBI patients were referred to our brachial plexus clinic, which serves as a tertiary treatment center for a population of 2 million people. All children were examined at birth by the referral centers’ pediatrician at a median age of 1 day (0–2) and at a median of 2 days (0–7) by a physiotherapist. Severity of the injury was classified at birth to global plexus injury or upper plexus injury (UP), UP meaning shoulder and elbow and in some patients wrist extension affected. Global plexus injury was further subclassified as flail upper extremity (FUE); no movement at all in the affected limb, complete plexus involvement (CP); shoulder, elbow, wrist, and hand affected. Presence of Horner’s sign was documented. Once referred to our clinic all patients were examined by a BPBI specialized team consisting of a hand surgeon, occupational therapist, and physiotherapist. Patients with persisting palsy were scheduled to be seen on regular basis by the same team at set time intervals from 1 month of age (at 3, 6, and 12 months, 2, 4, 7, 10, and 14 years of age). Active and passive range of motion (ROM) of all upper extremity joints were measured at each appointment using a goniometer. Muscle strength for shoulder abduction and flexion, elbow and wrist extension, and flexion, thumb and finger extension, and flexion was evaluated using the Medical Research Council’s scale for muscle strength. The 3-month Toronto Test Score was retrospectively calculated, since it was not used routinely in our institution during the study period.

MRI was scheduled for patients who had either GP at 1 month of age or UP with no antigravity biceps function by 3 months of age.

High-resolution cervical MRI (1.5T Philips Medical Systems, Achieva; Philips Healthcare, Best, Netherlands) was performed under general anesthesia. After localizer sequences, T1-weighted (T1-W) spin-echo images in sagittal plane were obtained. T2-weighted (T2-W) spin echo images were obtained in axial, sagittal, and coronal planes. Slice thickness was 2 mm for T2 coronal sequence and 3 mm for all other sequences. Heavily T2 weighted BFFE sequence in coronal and axial planes with 0.5 mm slice thickness allowed MR myelography view of the roots in each study. Type and number of root injuries (no avulsion, thinned roots, partial avulsion, and total avulsion) as well as location of pseudomeningoceles (PMC) were registered. Total root avulsion was defined as both anterior and posterior roots avulsed from the spinal cord. Partial avulsion was defined as either anterior or posterior root avulsed from the spinal cord. Thinned roots are seen on MRI when some of the rootlets emerging from the spinal cord, forming the anterior or posterior root, are ruptured (Silbermann-Hoffmann and Teboul 2013, Tse et al. 2014). T2-weighted axial sequences also covered both shoulders and therefore position (normal, posteriorly subluxated, posteriorly dislocated) of both humeral heads and the shape (normal, posteriorly rounded, pseudoglenoid) of both glenoids was recorded as well as the glenoscapular angle (GSA). A pediatric radiologist (TP), with more than 15 years of experience in musculoskeletal MRI, evaluated all images.

Brachial plexus exploration was recommended to all patients with total root avulsion(s) on MRI. If no total avulsion(s) were detected observation was continued for another 3 months. Surgery was then again recommended if no improvement was clinically observed. The length of time in days from MRI referral to MRI examination and to brachial plexus surgery was recorded. Both sensitivity and specificity for total avulsions and PMC on MRI was calculated in relation to the intraoperative findings. Findings in MRI and surgery were also compared with clinical outcome at a mean follow-up of 4.5 years (1.6–8.6) to assess given treatment. None of the patients were lost during follow-up.

### Statistics

Sensitivity and specificity for the MRI findings in comparison with the intraoperative findings as well as PMC in relation to root avulsion injury on MRI were calculated. The 95% confidence intervals (CI) were calculated using Wilson score intervals. Linear regression models were fitted for GSA difference and model assumptions were visually assessed.

Statistical analysis was done using R program for statistical computing (R Foundation for Statistical Computing, Vienna, Austria. R Core Team 2017). The significance level p < 0.05 was used.

### Ethics, funding, and potential conflicts of interest

The Ethics Committee of our hospital approved this study (registration number 79/E7/2001). Parental consent was gathered. No funding was received. No conflicts of interest were declared.

## Results

Inclusion criteria were met by 34/157 patients. Cervical MRI was performed on all 34 patients (18 boys) at median 3.9 months of age (0.3–14.3). Mean birth weight was 4,276 g (3,480–5,400). 22 of the injuries were on the right side. 1 of the 34 patients who had cervical MRI had a bilateral injury after breech delivery. 4 patients with an FUE at birth had a positive Horner’s sign.

Our diagnostic and treatment protocol could not be followed exactly as planned for patient, parent, and hospital related reasons. Children with persisting FUE or CP (n = 18) had the referral for MRI at median 2.1 month of age (0.2–3.5) and the MRI was performed at median 3.5 months of age (0.3–14.3) respectively. The respective ages of children with UP and no antigravity biceps function by 3 months of age (n = 16) were 3.0 months (0.9–6.2) and 4.0 months (1.7–7.7). Median time to MRI from referral was 28 days (1–70), excluding patient number 13 whose MRI was postponed twice (up to 328 days) for miscellaneous reasons. Based on the preliminary findings of this study 1 child (patient 32) with an FUE at birth was immediately referred for MRI.

**Figure 1.**
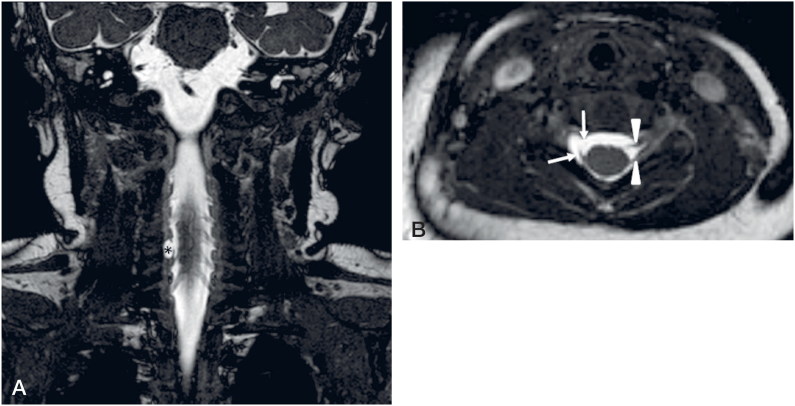
Coronal (A) and axial (B) BFFE MR images (0.5 mm) in a 3-month-old boy (patient 18) with brachial plexus birth injury on the right side. Total (both ventral and dorsal roots) avulsion of right C6 root with a PMC (asterisk): ventral root is avulsed from the cord (upper arrow), where a short stump of dorsal root is seen (lower arrow). Left ventral and dorsal C6 roots (arrowheads) are normal.

**Figure 2.**
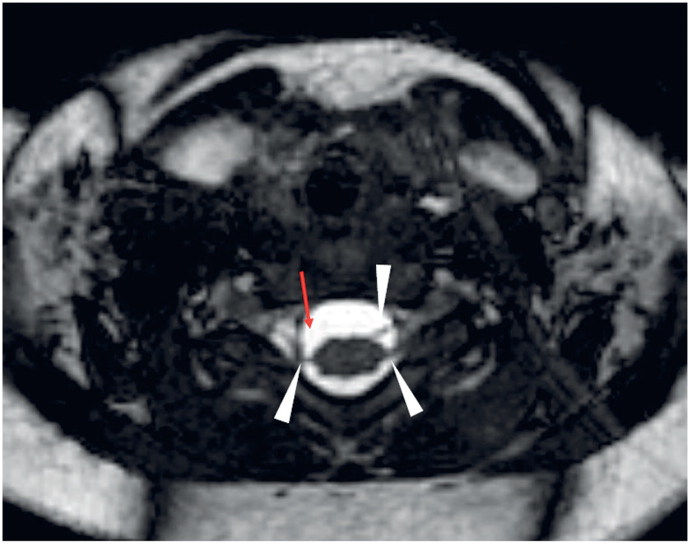
Axial BFFE MR image (0.5 mm) in a 4-month-old girl (patient 26) with brachial plexus birth injury on the right side. Partial avulsion of C8 root: ventral root is avulsed (red arrow), dorsal C8 root is intact (arrowhead). Left C8 nerve roots are normal (arrowheads).

**Figure 3.**
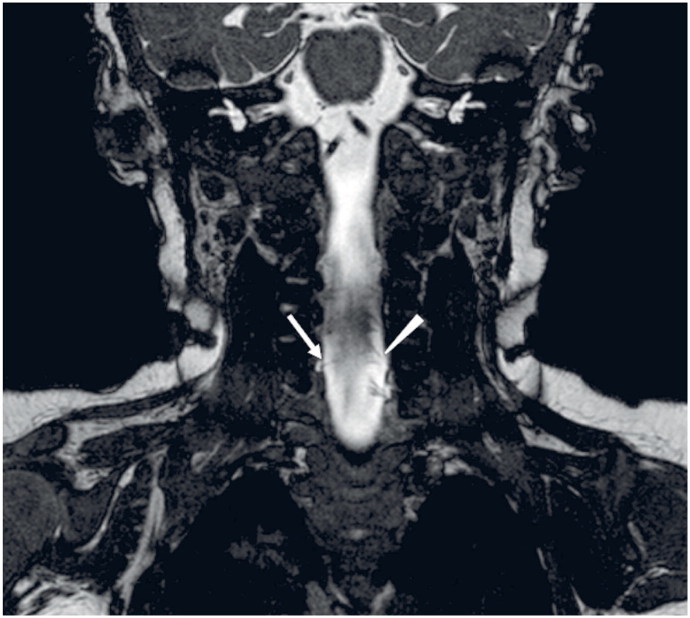
Coronal BFFE image (0.5 mm) in a 3-month-old girl (patient 16) with brachial plexus birth injury on the right side. Right ventral C6 root is thinned (arrow) compared to the normal left ventral C6 root (arrowhead).

**Figure 4.**
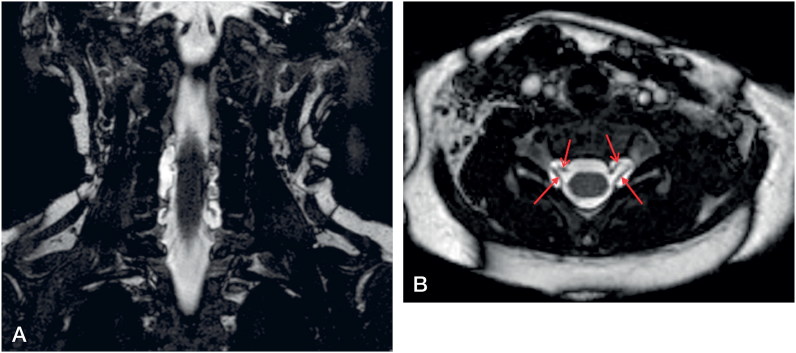
Coronal (A) and axial (B) BFFE image (0.5 mm) in 3-month-old boy (patient 31) with brachial plexus birth injury on both sides. (b) Intact ventral and dorsal nerve roots (red arrows) at C7 level despite a PMC clearly visible on both sides.

Altogether 170 root levels were examined. 18 total root avulsions were detected in 12/34 patients (Figure 1). 6 patients had partial avulsions only (dorsal root 2, ventral root 5) (Figure 2). 4 patients with total or partial root avulsions also had thinning of additional roots (Figure 3, Table 1). The most extensive injury was in patient number 8, who had total avulsions of C6–8 with thinning of both the ventral and dorsal C5 rootlets. The number of totally avulsed roots per patient varied from 1 to 3. The most commonly totally avulsed root was C8. Sensitivity and specificity of MRI in detecting total root avulsions was 0.88 (CI 0.5–1) and 1 (CI 1–0.9). PMC was seen in association with all 18 total root avulsions, and in 6 of the 8 partial avulsions at the level of the avulsion. 2 patients had PMC without evidence of root injuries (Figure 4). Specificity and sensitivity of PMC for total nerve root avulsion was 0.44 and 1.

**Table 1.** Patient demographics. Patients are arranged in descending order of abnormal findings on MRI

Findings at birth		MRI findings^b^					Plexus reconstruction			
Patient	Extent of injury^a^	3-month test score	Age (months)	Total root avulsion	Partial root avulsion	Thinning of roots	PMC	Age (months)	Plexus reconstruction	Converted to SAN pro SSN
8	FUE^c^	0	0.9	C6–8		C5vd	C5–8	6.6	CC7	
25	FUE	0	2.7	C8–T1			C8–T1	4.3	yes	
15	FUE^c^	0.3	2.6	C8–T1			C8–T1	3.4	yes	
34	CP	2.1	2.5	C7–8			C7–8		refused	
22	CP	2.8	3.2	C7–8			C7–T1	7.4		yes
3	FUE	0	4.0	C8			C8		refused	
32	FUE^c^	^d^	0.3	C8			C7–T1	0.8	yes	
9	FUE	0	3.4	C8			C8	4.4	yes	
20	FUE	1.3	4.0	C8	C7D		C8–T1	5.6	yes	
24	CP	1.8	3.7	C8			C8	5.7	yes	
13	FUE	2.6	14.3	C7			C7	27.9		yes
18	CP	3.2	3.0	C6			C6	7.3	yes	
17	FUE	0.6	1.9					3.9	yes	
11	CP	1.2	4.6					6.6	yes	
21	FUE^c^	1.3	3.9					9.0	yes	
1	CP	2.1	6.4		C6V, C8V		C6 8		refused	
26	CP	2.4	4.5		C8V	C7vd	C8	5.6	yes	
10	CP	3.8	3.3		C8V		C8			
14	UP	4.8	7.1		C6V		C6	8.0		yes
28	UP	4.5	3.9		C6D	C6v	C6			
16	CP	4.8	3.9		C6D	C6v				
31	UP	3.8	3.5				C5 6 7			
19	UP	4.8	7.7				C8			
27	CP	2.5	3.9					4.8		yes
29	CP	4.2	3.2							
23	CP	4.5	4.4							
6	UP	4.8	3.2							
33	UP	5.2	4.3							
30	CP	5.2	3.9							
2	CP	5.5	4.6							
7	UP	5.8	3.4							
4	UP	5.8	4.7							
5	UP	5.8	4.1							
12	UP	5.8	1.7							

a FUE = Flail upper extremity, CP = Complete plexus involvement, UP = Upper plexus involvement,

b V = Ventral root, D = Dorsal root, v = Ventral root thinning, d = Dorsal root thinning, SAN = Spinal accessory nerve, SSN = Supra-

scapular nerve.

c Horner sign.

d Primary surgery before 3 months of age

**Figure 5.**
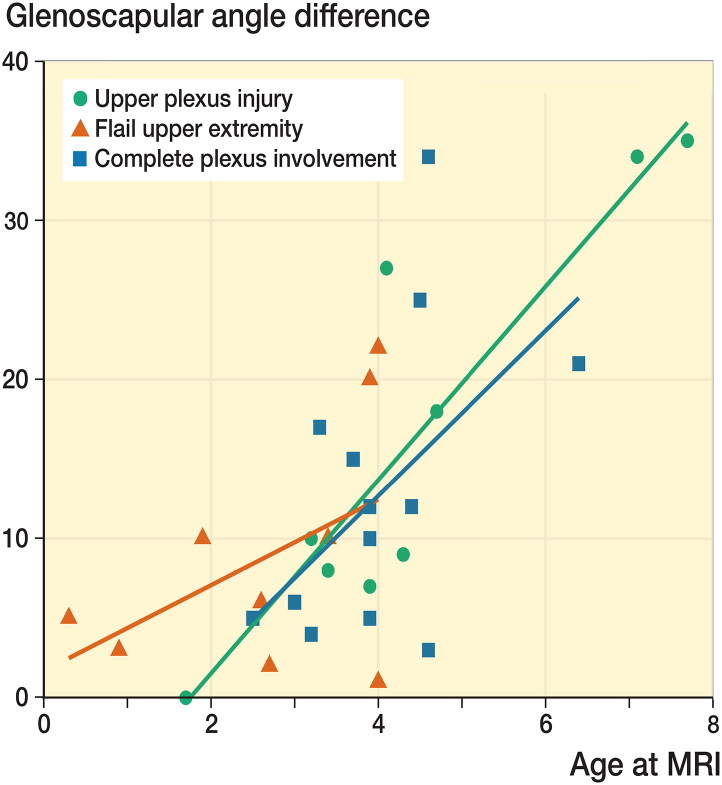
Multivariable model expressing GSA difference in relation to age at time of MRI and clinical findings at birth.

Asymmetry (> 5°) in GSA was recorded in 22 patients with a mean difference of 17° (6–35) (Table 2). GSA difference was modelled using linear regression with findings at birth and age at MRI as the covariates. Patient 13 was excluded due to significant delay until MRI. Both univariable and multivariable models were fitted. The findings at birth did not statistically significantly associate with the GSA difference in either the univariable or the multivariable models (p > 0.05 for both FUE and CP when compared with UP in both models). The age at MRI was associated significantly with GSA difference in both models; 4.7 (CI 3–6.5) per year in univariable and 5 (CI 3–7) in the multivariable model, p < 0.001 in both cases (Figure 5). Glenoid shape was normal in 20 patients, with a trend towards more severe incongruence in the patients with an older age at MRI.

**Table 2.** Glenohumeral joint (GHJ) MRI findings. Patients are arranged primarily in descending order based on incongruency of their affected shoulder, secondarily in descending order based on the difference between GSA of both shoulders

Patient	Findings at birth^a^	MRI age (months)	GHJ	Glenoid shape	GSA (°)affected	GSA (°)normal	GSA difference
19	UP	7.7	D	PG	–40	–5	35
11	CP	4.6	D	PG	–40	–6	34
14	UP	7.1	D	PG	–40	–6	34
5	UP	4.1	D	PG	–30	–3	27
3	FUE	4.0	D	PG	–25	3	22
10	CP	3.3	D	PG	–25	–8	17
16	CP	3.9	D	PG	–25	–20	5
26	CP	4.5	SL	PR	–40	–15	25
1	CP	6.4	SL	PR	–30	–9	21
21	FUE	3.9	SL	PR	–25	–5	20
4	UP	4.7	SL	PR	–40	–22	18
24	CP	3.7	SL	PR	–25	–10	15
9	FUE	3.4	SL	PR	–30	–20	10
6	UP	3.2	SL	PR	–25	–15	10
30	CP	3.9	SL	N	–20	–10	10
27	CP	3.9	N	N	–15	–3	12
23	CP	4.4	N	N	–20	–8	12
17	FUE	1.9	N	N	–20	–10	10
33	UP	4.3	N	N	–20	–11	9
7	UP	3.4	N	N	–20	–12	8
28	UP	3.9	N	N	–15	–8	7
15	FUE	2.6	N	N	–13	–7	6
28	CP	3.0	N	N	–13	–7	6
32	FUE	0.3	N	N	–15	–10	5
34	CP	2.5	N	N	–15	–10	5
22	CP	3.2	N	N	–10	–6	4
29	CP	3.2	N	N	–5	–1	4
8	FUE	0.9	N	N	–9	–6	3
13	FUE	14.3	N	N	–7	–10	3
2	CP	4.6	N	N	–8	–5	3
25	FUE	2.7	N	N	–5	–7	2
20	FUE	4.0	N	N	–5	–4	1
12	UP	1.7	N	N	–5	–5	0
31	UP/UP	3.5	N/N	N/N	–15/–20		
							

a See Table 1.

D = Dislocated, SL = Subluxed, N = Normal, PG = Pseudoglenoid,

PR = Posteriorely rounded

Reconstructive nerve surgery was recommended to all 12 patients with total avulsions on MRI and to 7 of the 22 patients without total avulsions. Parents consented to surgery in 10 patients with total avulsions on MRI (reconstruction with autologous nerve grafts 7, spinal accessory nerve (SAN) pro suprascapular nerve (SSN) transfer 2, contralateral C7 transfer 1), and 6 patients without total avulsions respectively (reconstruction with autologous nerve grafts 4, SAN pro SSN transfer 2). Median age at primary reconstructive nerve surgery was 5.6 months (0.8–9) excluding patient number 13 who was operated at 28 months of age. According to the retrospective calculation of the 3-month Toronto Test Score, 18 of the 19 patients for whom we recommended plexus surgery had a Test Score less than 3.5, which is an indication for plexus reconstruction (Borschel and Clarke 2009) (Table 1).

Intraoperative findings concerning total avulsions were compared with corresponding findings on MRI (Table 3). Sensitivity and specificity of MRI in detecting total nerve root avulsions was 0.88 and 1. Three total C8 avulsions, one accompanied by a total C7 avulsion, were left unexplored due to good hand and wrist function at the time of surgery (Table 3). Median time from MRI to primary surgery was 49 days (13–173) excluding patient number 13 whose MRI and operation were delayed.

**Table 3.** MRI findings compared to intra-operative findings by root level

	C5		C6		C7		C8		T1			PMC
Patient	MRI	Surgery	MRI	Surgery	MRI	Surgery	MRI	Surgery		MRI	Surgery	MRI
9	N	N	N	N	N	N	A	A		N	—	Yes
11	N	N	N	N	N	—	N	—		N	—	No
15	N	N	N	N	N	N	A	A		A	A	Yes
17	N	N	N	N	N	N	N	N		N	—	No
18	N	N	A	A	N	N	N	—		N	—	Yes
20	N	N	N	N	D	—	A	—		N	—	Yes
21	N	N	N	N	N	N	N	—		N	—	No
24	N	N	N	N	N	N	A	—		N	—	Yes
25	N	N	N	N	N	N	A	A		A	A	Yes
26	N	N	N	N	v + d	N	V	N		N	—	Yes
32	N	N	N	N	N	A	A	A		N	—	Yes

N = Normal, A = Avulsion, V = Ventral root avulsion, D = Dorsal root avulsion,

v = Ventral root thinning, d = Dorsal root thinning, — = Not examined

True positive was defined as a total root avulsion seen on MRI and detected during exploration. True negative was defined as no avulsion seen on MRI nor detected during surgery. Partial and thinned roots seen on MRI as well as roots not explored during surgery were left out of the calculation.

None of the 34 patients recovered completely during follow-up. Additional surgery was performed in 4 patients with total root avulsions, 1 patient with partial root avulsions, and 9 patients without root injuries (Table 4).

**Table 4.** Patient outcome. Outcome expressed as ratio (%) of active antigravity range of motion of the affected side in comparison with the unaffected side. Patients are arranged primarily by the extent of injury at birth and secondarily by their 3-month Test Score

Outcome													
Patient	Findings at birth ^**a**^	3-month test score	Plexus surgery ^d^	Nerve transfer	GHJ relocation	Other surgery	A	B	C	D	E	F	G
8	FUE ^b^	0	CC7		yes	EIP > EPB,	8.0	CP	91	0	33	0	0
3	FUE	0	refused	SAN > AN		BR > EDC	8.6	CP	28	73	17	50	0
32	FUE ^b^	^c^	5 > 8, 6 > UT				2.6	CP	50	38	25	25	0
9	FUE	0	5–6 > UT, SAN > SSN	SAN > ISN	yes		7.3	CP	33	92	10	10	10
25	FUE	0	5 > 6, 6 > 1, 7 > 8, SAN > SSN			forearm	3.7	CP	36	50	10	40	20
						osteotomy							
15	FUE ^b^	0.3	5 > UT, 6 > MT, 7 > 81,				4.5	CP	50	63	72	0	10
			SAN > SSN										
17	FUE	0.6	5–6 > UT, 7 > MT, SAN > SSN		yes		5.5	CP	50	75	11	20	10
11	CP	1.2	5–6 > UT, SAN > SSN				6.2	UP	44	28	61	100	100
21	FUE ^b^	1.3	5–6 > UT, 7 > MT, SAN > SSN	Oberlin		FCU > ECR	5.0	CP	50	84	17	50	50
20	FUE	1.3	5–6 > UT, SAN > SSN			TM > IS	4.5	UP	78	88	100	100	100
24	CP	1.8	5 > UT, 6 > UT, 7 > MT,	Oberlin			4.2	UP	50	80	100	100	100
			SAN > SSN		yes								
1	CP	2.1	refused				5.2	UP	40	100	100	100	100
34	CP	2.1	refused				1.6	UP	38	38	25	100	100
26	CP	2.4	6 > UT, SAN > SSN				3.2	UP	44	62	100	100	100
27	CP	2.5	converted to nerve transfer				3.0	UP	78	81	100	100	100
13	FUE	2.6	converted to nerve transfer	SAN > SSN			5.6	UP	39	63	56	100	100
				SAN > SNN,									
22	CP	2.8	converted to nerve transfer	pRN > pAN			4.0	UP	38	75	61	100	100
18	CP	3.2	5 > 6, SAN > SSN	SAN > SSN			5.0	UP	83	88	100	100	100
10	CP	3.8					6.2	UP	69	81	100	100	100
31	UP	3.8		SAN > ISN			2.9	UP	89	81	100	100	100
29	CP	4,2		SAN > ISN			3.5	UP	72	81	100	100	100
28	UP	4.5		SAN > ISN			2.2	UP	89	97	100	100	100
23	CP	4.5					2.9	UP	89	91	100	100	100
14	UP	4.8	converted to nerve transfer				6.1	UP	66	90	100	100	100
6	UP	4.8		SAN > SSN	yes		5.2	UP	94	100	100	100	100
19	UP	4.8					4.5	UP	40	56	56	100	100
16	CP	4,8					4.0	UP	88	91	100	100	100
33	UP	5.2					2.4	UP	81	81	100	100	100
30	CP	5.2		SAN > ISN			3.2	UP	92	100	100	100	100
2	CP	5.5		SAN > ISN	yes	TM > IS	7.4	UP	60	87	100	100	100
7	UP	5.8			yes	TM > IS	6.9	UP	50	71	36	100	100
4	UP	5.8					2.1	UP	72	69	100	100	100
5	UP	5.8					8.4	UP	94	81	100	100	100
12	UP	5.8					4.8	UP	89	100	100	100	100
													

**^a^**See Table 1.

**^b^**positive Horner sign

**^c^** primary surgery before 3 months of age

**^d^**AN = Axillary nerve, pAN = partial axillary nerve, BR = Brachioradialis muscle, CC7 = contralateral C7 transfer, ECR = Extensor carpi radialis longus and brevis muscle, EDC = Extensor digitorum communis muscle, EIP = Extensor indicis proprius muscle, EPB = Extensor pollicis brevis muscle, FCU = Flexor carpi ulnaris muscle, IS = Infraspinatus muscle, ISN = Infraspinatus branch of suprascapular nerve, M = Middle trunk, pRN = partial Radial nerve, SAN = Spinal accessory nerve, SSN = Suprascapular nerve, TM = Teres major muscle, UT = Upper trunk

Retrospectively, assessed by final outcome (Table 4) expressed by ratios (injured vs. uninjured side) of active antigravity shoulder, elbow, wrist, and finger ROM, all patients who had total root avulsions on MRI and all children born with FUE would have benefited from plexus surgery. On the other hand, based on the final outcome (Table 4), 2 (patients 7 and 19) of the 10 patients with upper plexus palsy at birth might have benefited from plexus reconstruction. Retrospectively analyzed, both of these patients would have failed the Cookie Test at 9 months. When looking at the patient final outcome, partial root avulsion alone, or in combination with thinned rootlets (6 patients), had no clinical significance (Table 4).

## Discussion

Clinical evaluation of the extent and type of root injuries in BPBI forms the basis for indication, timing, and planning of surgical repair. Distinction of BPBI patients with axonotmesis type of root injuries, with potential for spontaneous recovery, from infants with root ruptures and/or avulsions that usually benefit from surgical treatment, however, remains a challenge for brachial plexus surgeons. Our aim was to find out whether cervical MRI could be helpful in surgical decision-making in patients with permanent BPBI.

CT myelography has long been the gold standard in BPBI diagnostic imaging, but during recent years there has been a clear trend towards MRI, possibly due to the fact that MRI does not involve ionizing radiation or the need for intrathecal contrast injection. Earlier MRI studies with evaluation of the presence of PMC only (Tse et al. 2014) or of nerve root integrity with 1.5 mm MRI slice thickness (Medina et al. 2006) have demonstrated only moderate sensitivity or specificity levels for root avulsions. In contradiction to these earlier reports we found an excellent correlation between complete root avulsions and surgical findings using 1.5 T MRI with 0.5 mm slice thickness in axial and coronal views. Sensitivity and specificity for complete root avulsion on MRI in our study are in line with the more recent studies of Somashekar et al. (2014) and Menashe et al. (2015). Our study further confirmed that PMC has a high sensitivity but low specificity for total nerve root avulsions on MRI (Yilmaz et al. 1999, Medina et al. 2006). We did not explore all levels that showed root avulsion on MRI and thus left these unconfirmed findings outside the sensitivity and specificity calculation.

We also found that evidence of total root avulsion(s) on MRI in itself was a good indicator for brachial plexus exploration and reconstruction. The clinical findings did not always correlate with a complete C8 avulsion on MRI. Hand recovery could therefore not be reliably predicted by MRI in our patients since functional recovery of the hand and wrist were good in some without exploration and surgical repair of C8, despite it appearing completely avulsed on the MRI. These patients underwent surgical reconstruction of the upper plexus, which appeared to be beneficial assessed by the final outcome. Our MRI protocol also enabled imaging of thinned rootlets and partial root avulsions. Their existence did not appear to influence the outcome negatively, which is why it is probably better to leave roots that show evidence of partial avulsion or thinning on MRI unexplored.

As of today, there have been no studies published regarding the use of 3T MRI for root diagnostics in infants with BPBI. Further studies are needed to establish the role of 3T MRI compared with 1.5 T MRI concerning root avulsion diagnostics in BPBI.

FUE with or without a positive Horner’s sign at birth was a good indicator for a difficult permanent injury and plexus reconstruction. All 10 patients in this series with an FUE at birth had surgery. Root avulsions were evident in 8/10, and neuromas in continuity in all 10 of these children. This is in line with previous findings by Grossman et al. (2004), Hale et al. (2010) and Abid et al. (2016), and is the reason why in our practice we have turned towards very early surgical exploration in patients with FUE. Some surgeons prefer to use the 3-month Toronto Test Score when evaluating the need for surgery (Borschel and Clarke 2009) and in fact this method gave concurrent recommendations for surgery in 33 of the 34 patients in our study. 1 patient with a test score of 4.8 was scheduled for plexus reconstruction, but the operation was converted to nerve transfer at the time of surgery due to better than expected recovery (patient 14). In addition, preoperative clinical re-evaluation converted plexus reconstruction to nerve transfer for 3 more patients. Later analysis of these patients revealed that the decision for conversion was appropriate for 1 patient (patient 27) but 2 patients would most likely have benefited from plexus reconstruction (patients 13 and 22). 2/10 of our patients with UP at birth might have benefited from upper plexus reconstruction or extraplexal neurotization procedures since they did not reach above horizontal shoulder abduction, with elbow and wrist movement also clearly compromised. Retrospectively we found that both of these patients had failed the Cookie Test at 9 months of age. Thus, postponing the decision to do surgery in patients with no avulsions on MRI, but a Toronto Test Score at 3 months below 3.5, or in patients that fail the Cookie Test at 9 months is controversial.

Untreated posterior shoulder subluxation in BPBI leads to permanent reduction of shoulder ROM and glenohumeral deformity (Hoeksma et al. 2003, Pöyhiä et al. 2007). Maintenance of good passive shoulder ROM, treatment of posterior subluxation with Botulinum Toxin A injections and early surgical reduction of the shoulder may prevent these adverse shoulder sequelae in BPBI (El-Gammal et al. 2006, Ezaki et al. 2010, Pöyhiä et al. 2011). Shoulder subluxation proceeds gradually to dislocation, which was evident also in our study where the severity of changes in shoulder congruency correlated to patient age. First signs of glenohumeral joint incongruence were recognized already under 2 months of age in some of our patients. This is in accordance with the earlier study of Pöyhiä et al. (2010) where half of the patients with permanent BPBI and shoulder pathology had already developed posterior shoulder subluxation at 3 months of age.

Our study has limitations despite its prospective nature. First, the sample size is relatively small; second, our patient population is heterogeneous and third, all totally avulsed roots on MRI were not surgically explored. Therefore further studies with more patients are needed to verify our main findings: FUE at birth, total root avulsions on MRI, and/or a 3-month Test Score < 3.5 are good indicators for brachial plexus exploration and reconstruction in BPBI.

PG: Main author, hand surgeon. Part of brachial plexus birth injury team. Clinical work and development of study protocol. TP: Second author, pediatric radiologist, and developer of the imaging protocol. Part of brachial plexus injury team. AS: Co-author, hand surgeon. Part of brachial plexus birth injury team. Clinical work and development of study protocol. YN: Senior author. Lead of brachial plexus birth injury team. Clinical work and development of study protocol.

## Abbreviations

A.: Final follow-up age (years)
B.: Findings at final follow-up: CP = Complete plexus involvement, UP = Upper plexus involvement
C.: Shoulder abduction (%)
D.: Elbow flexion (%)
E.: Wrist ex-tension-flexion (%)
F.: Finger movement (%)
G.: Intrinsic (%)
